# Porous Organic Polymers Derived from Ferrocene and Tetrahedral Silicon-Centered Monomers for Carbon Dioxide Sorption

**DOI:** 10.3390/polym14030370

**Published:** 2022-01-18

**Authors:** Xingya Zhao, Yipeng Qi, Jianquan Li, Qingyu Ma

**Affiliations:** 1 School of Materials Science and Engineering, University of Jinan, Jinan 250022, China; zhaoxingyaya@gmail.com (X.Z.); qiyipeng@hnksac.com (Y.Q.); mse_lijq@ujn.edu.cn (J.L.); 2 College of Chemistry, Chemical Engineering and Materials Science, Shandong Normal University, Jinan 250014, China; 3 Department of Chemistry, University of Waterloo, Waterloo, ON N2L 3G1, Canada

**Keywords:** porous organic polymers, ferrocene, silicon-centered monomers, carbon dioxide sorption, gas storage

## Abstract

Herein, we present two novel ferrocene-containing porous organic polymers, FPOP-1 and FPOP-2, by the Heck reactions of 1,1′-divinylferrocene with two tetrahedral silicon-centered units, i.e., tetrakis(4-bromophenyl)silane and tetrakis(4′-bromo-[1,1′-biphenyl]-4-yl)silane. The resulting materials possess high thermal stability and moderate porosity with the Brunauer–Emmer–Teller (BET) surface areas of 499 m^2^ g^−1^ (FPOP-1) and 354 m^2^ g^−1^ (FPOP-2) and total pore volumes of 0.43 cm^3^ g^−1^ (FPOP-1) and 0.49 cm^3^ g^−1^ (FPOP-2). The porosity is comparable to previously reported ferrocene-containing porous polymers. These materials possess comparable CO_2_ capacities of 1.16 mmol g^−1^ (5.10 wt%) at 273 K and 1.0 bar, and 0.54 mmol g^−1^ (2.38 wt%) at 298 K and 1.0 bar (FPOP-1). The found capacities are comparable to, or higher than many porous polymers having similar or higher surface areas. They have high isosteric heats of up to 32.9 kJ mol^−1^, proving that the affinity between the polymer network and CO_2_ is high, which can be explained by the presence of ferrocene units in the porous networks. These results indicate that these materials can be promisingly utilized as candidates for the storage or capture of CO_2_. More ferrocene-containing porous polymers can be designed and synthesized by combining ferrocene units with various aromatic monomers under this strategy and their applications could be explored.

## 1. Introduction

Porous organic polymers (POPs) are a specific class of covalently bonded functional materials containing permanent porosity and constructed from organic monomers through various synthetic methodologies. By virtue of their diversified functionality (e.g., porosity, fluorescence, ionic charges), POP materials have found extensively potential applications in gas separation, gas storage, capture of pollutants, optoelectronics, energy storage, catalysis, sensing, nanoreactors, and medical applications [1,2,3,4,5,6]. Among various POP materials, the metal atoms containing POPs have gained particular interest as the incorporation of metal sites into the porous networks could impart the resultant materials new functions (e.g., catalytic activity and fluorescence), and thus extend/enhance their application performance (e.g., enhanced CO_2_ adsorption) [7,8,9,10,11,12,13,14,15,16]. For example, Son et al., reported a mesoionic carbene-Rh species containing microporous organic polymer exhibiting high catalytic activity in the stereoselective polymerization of arylacetylenes by the functionalization of azide-alkyne click-based microporous polymer [9]. The vanadium-docked covalent organic frameworks have served as efficient heterogeneous catalysts for Mannich-type reactions [17]. By doping alkali-metal into POP networks, the CO_2_ and H_2_ uptakes could be efficiently improved due to the enhanced isosteric heat of gas adsorption values [8]. At present, most of them were prepared by post-synthetic modification, i.e., by post metalation treating with pre-synthesized POPs containing coordinated atoms (e.g., N, S) or functional groups [8,18,19,20,21,22]. However, the successful achievement of these materials requires at least two steps. In addition, the modification efficiency of metal sites or nanoparticles on the frameworks may be not very high due to the heterogeneous characteristic, and the metal sites may also aggregate in the networks. It could be expected that the direct synthetic strategy based on predesigned monomers containing metal sites is more convenient by selecting various polymerization reactions, including the Sonogashira–Hagihara reaction, Suzuki reaction, Yamamoto reaction, and Friedel–Crafts reaction, etc. [1,2,23]. Furthermore, this strategy can lead to regular and homogeneous dispersion of metal sites in the networks, thus allowing for enhanced interactions between the frameworks and guest analytes (e.g., gas) and higher performance [24].

Ferrocene as a typical metallocene has been widely introduced into polymers to construct novel functional materials and extend their applications, such as magnetism, sensors, electrocatalysis, and medicine, thanks to its unique sandwich structure with special functionality [10,25,26,27]. Recently, ferrocene was also incorporated into POP networks and endow them new applications or enhanced performance. For example, Liu et al., reported ferrocene-based conjugated microporous polymers with intrinsic magnetism and the saturation magnetization of the materials can be adjusted by combining ferrocene with monomers with different conjugated degree [28]. By taking advantage of abundant ferrocene with high electron density and strong interaction with iodine molecules, a ferrocene-containing porous network FcTz-POP exhibited a significantly higher iodine vapor capacity than a ferrocene-free POP [29]. FcTz-POP can also served as a photocatalyst for methylene blue degradation under visible light irradiation at neutral pH [30]. By virtue of the strong interaction between the iron sites and gas molecules, Fu et al., constructed ferrocene-functionalized microporous aromatic polymers with a moderate porosity (BET surface area, 875 m^2^ g^−1^) and good gas sorption performance by the Friedel–Crafts reaction of ferrocene and *s*-triazine monomers [24]. Liu et al., reported ferrocene-based conjugated microporous polymers exhibiting good gas storage capacity and excellent methyl violet adsorption by the Yamamoto reactions of 1,10-dibromoferrocene and 5,10,15,20-tetrakis(4-bromophenyl)porphyrin or tetra(*p*-bromophenyl)methane [31]. Although some ferrocene-based POPs have been reported, compared to the abundant porous materials, the construction novel ferrocene-containing POP materials and their applications are far from fully explored [32,33,34,35,36]. The design and construction of novel ferrocene-based POPs is still desirable.

In the present study, we synthesized two novel ferrocene-containing porous organic polymers (FPOPs) based on 1,1′-divinylferrocene and tetrakis(4-bromophenyl)silane or tetrakis(4′-bromo-[1,1′-biphenyl]-4-yl)silane by the Heck reaction. The resulting polymers show high thermal stability and moderate porosity. Moreover, the application in CO_2_ adsorption was also investigated.

## 2. Materials and Methods

### 2.1. Materials and Characterization

Tetra(phosphorylation)palladium (Pd(PPh_3_)_4_) and potassium carbonate (K_2_CO_3_) were purchased from Energy Chemical. *N*,*N*-Dimethylformamide (DMF) was bought from Tianjin Kemiou Chemical Reagent Co., Ltd. (Tianjin, China). DMF was dehydrated with CaH_2_ at 80 °C for 12 h, distilled under vacuum, and stored with 4 Ǻ molecule sieves prior to use. 1,1′-Divinylferrocene, tetrakis(4-bromophenyl)silane, and tetrakis(4-bromobiphenyl)silane were prepared based on the methods in previous reports [37,38,39].

### 2.2. Characterization

Fourier-transform infrared (FT-IR) spectra were recorded on a Bruker Tensor 27 spectrophotometer (Bruker, Ettlingen, Germany). The solid-state ^13^C cross-polarization/magic-angle-spinning (CP/MAS) NMR and ^29^Si MAS NMR spectra were measured by a Bruker AVANCE-500 NMR Spectrometer, while the operating magnetic field strength is 9.4 T. The field-emission scanning electron microscopy (FE-SEM) images was observed by a FEI QUANTA FEG250 Spectrometer (FEI, Hillsboro, OR, USA). Before observation, the samples were prepared by dropping the suspensions in ethanol on a copper grid and following air drying. The thermogravimetric analysis (TGA) was determined by a MettlerToledo SDTA-854 TGA system (Greifensee, Switzerland). The samples were heated at a rate of 10 °C min^−1^ in the range of room temperature to 800 °C in an atmosphere of nitrogen.

Nitrogen sorption isotherms were measured at 77 K with a Micromeritics ASAP 2020 (Norcross, GA, USA) volumetric adsorption analyzer. Prior to the measurement, the samples of ca. 100 mg were degassed in vacuum at 150 °C for 12 h. The specific surface areas were calculated based on the nitrogen adsorption data by Brunauer–Emmett–Teller (BET) analysis in the relative pressure (P/P_0_) range of 0.01~0.20, whereas the pore size distribution was evaluated on the basis of nonlocal density functional theory (NL-DFT). The total pore volumes were calculated at P/P_0_ = 0.99, whereas the micropore surface area and micropore volume were calculated using the *t*-plot method. The carbon dioxide (CO_2_) adsorption isotherms were measured on a Micrometrics ASAP 2020 volumetric adsorption analyzer at 298 K and 273 K. Before the measurements, the samples were degassed in vacuum at 150 °C for at 12 h.

### 2.3. Synthesis of FPOP-1

In a three-necked bottle, 1,1′-divinylferrocene (0.24 g, 1 mmol), tetrakis(4-bromophenyl)silane (0.33 g, 0.5 mmol), K_2_CO_3_ (0.55 g, 4 mmol), Pd(PPh_3_)_4_ (0.12 g, 0.10 mmol), and 20 mL of freshly distilled DMF were added under an atmosphere of argon. The argon was bubbled into the mixture for at least 0.5 h to remove oxygen in the system. Then, the mixture was heated to 120 °C and kept for 72 h under argon. The resultant mixture was cooled to room temperature and filtered with a vacuum equipment. The resultant precipitate was washed with various solvents, including water, methanol, chloroform, tetrahydrofuran (THF), and acetone to remove the residuals. The obtained crude product was further purified by Soxhlet extraction with methanol and THF for 24 h, respectively. Finally, the product, which was denoted as FPOP-1, was dried in a vacuum oven at 80 °C for 24 h and appeared as a red-brown solid (213 mg, yield: 53.0%). Elemental analysis calc. (wt%) for SiC_52_H_36_Fe_2_: C 78.2, H 4.3; found C 72.5, H 4.0.

### 2.4. Synthesis of FPOP-2

The synthetic and post-treatment procedures of FPOP-2 were similar to those of FPOP-1 except that tetrakis(4′-bromo-[1,1′-biphenyl]-4-yl)silane (0.48 g, 0.5 mmol) was used as the starting material instead of tetrakis(4-bromophenyl)silane. FPOP-2 was obtained as a red-brown powder (324 mg, yield: 50.1%). Elemental analysis calc. (wt%) for SiC_76_H_48_Fe_2_: C 82.9, H 4.4; found C 80.7, H 4.5.

## 3. Results

### 3.1. Synthesis and Characterization

**Scheme 1:**
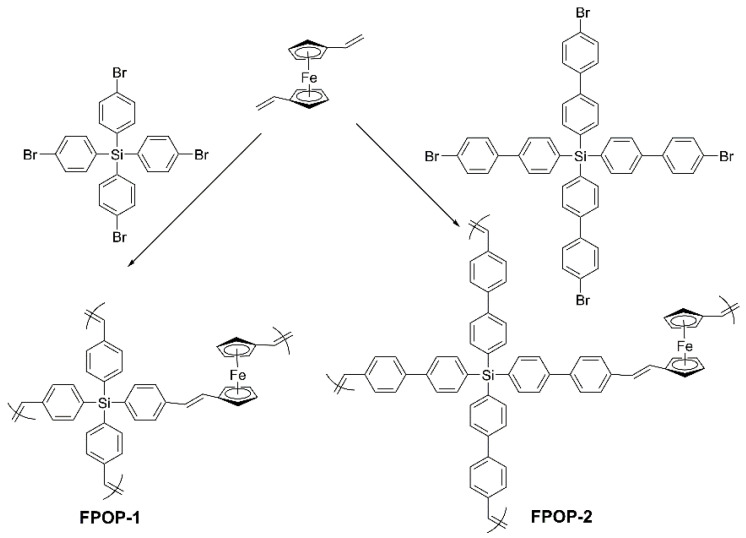
Synthesis routes of FPOP-1 and FPOP-2. Reaction condition: Pd(PPh_3_)_4_, DMF, and K_2_CO_3_, at 120 °C for 72 h. The fragments of FPOPs are shown as examples.

The synthetic routes of two novel ferrocene-based porous organic polymers are shown in Scheme 1. FPOP-1 and FPOP-2 were prepared by the Heck coupling reactions of 1,1′-divinylferrocene with two tetrahedral silanes, i.e., tetrakis(4-bromophenyl)silane and tetrakis(4′-bromo-[1,1′-biphenyl]-4-yl)silane. The reactions were conducted using Pd(PPh_3_)_4_ as the catalyst and K_2_CO_3_ as the acid absorbent in DMF at 120 °C for 72 h [40]. Upon the completion of the reaction, the crude products were filtrated and washed with various solvents to remove the residuals, including unreacted monomers, residual catalysts, and other impurities in the final products. Subsequently, the products were further purified by Soxhlet extraction and dried in a vacuum at 80 °C for 24 h. The final materials were afforded as red-brown powders. As expected, these polymers are insoluble in common solvents such as tetrahydrofuran, water, ethanol, and DMF.

**Figure 1:**
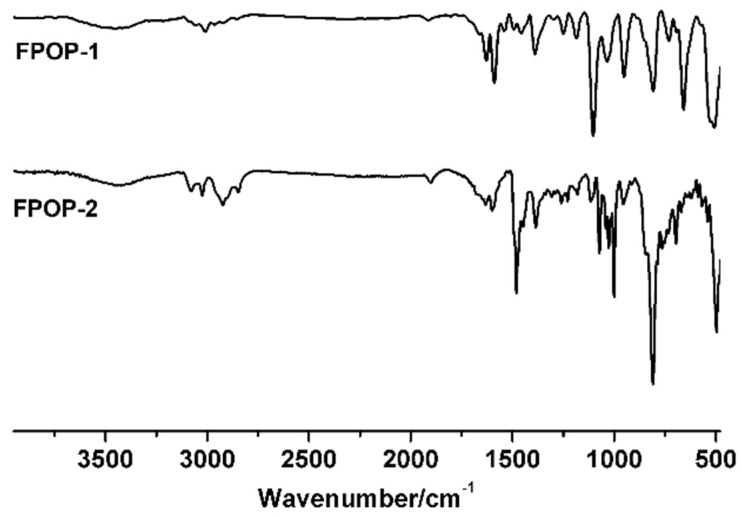
FT-IR of FPOP-1 and FPOP-2.

To confirm the structures of these polymers, they were fully characterized by FT-IR, solid-state ^13^C CP/MAS NMR, and ^29^Si NMR spectroscopy. FPOP-1 and FPOP-2 display similar spectra due to their similar chemical structures. For FT-IR spectra as shown in Figure 1, the multiple peaks from 1600 cm^−1^ to 1400 cm^−1^ are obviously attributed to the C=C stretching vibrations from phenyl and cyclopentadiene rings in tetrahedral silicon-centered units and ferrocene units. The peaks ranging from 3000 cm^−1^ to 3100 cm^−1^ are apparently associated to the unsaturated C–H stretching vibrations from phenyl and cyclopentadiene rings. In addition, two kinds of unexpected peaks were observed at ca. 3450 cm^−1^ and 2900–3000 cm^−1^. The former was attributed to –OH groups from water encapsulated in the samples, while the latter was aroused from saturated C–H groups, which are from the residual solvents (e.g., THF and methanol) in the network during the Soxhlet extraction process. Although the samples have been dried under vacuum, it is very difficult to completely remove all the residual solvents, including water, THF and methanol, because some of them may be embedded in the closed pores. This finding is consistent with our previous report [41].

**Figure 2:**
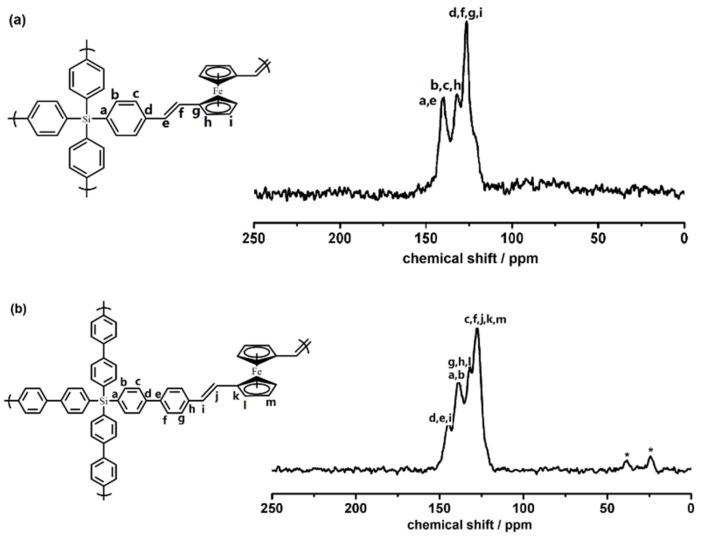
Solid-state ^13^C CP/MAS NMR spectra of FPOP-1 (**a**) and FPOP-2 (**b**). The peak indicated by asterisk may be assigned to the carbon atom from residual methanol resulting from the Soxhlet extraction.

**Figure 3:**
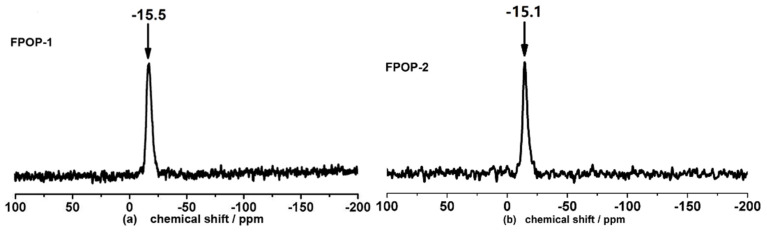
Solid-state ^29^Si NMR spectra of FPOP-1 (**a**) and FPOP-2 (**b**).

Figure 2 shows the solid-state ^13^C CP/MAS NMR spectra of these materials and the resonances were assigned. As expected, the peaks attributable to *sp*^2^ carbon atoms from ethenylene, phenylene and cyclopentadiene groups in ferrocene and silicon-centered units were observed with overlapping from 120 ppm to 150 ppm. For ^29^Si NMR spectra, both of FPOP-1 and FPOP-2 display one peak at −15.5 ppm and −15.1 ppm, respectively (Figure 3), which are unambiguously derived from the Si atoms from tetrahedral Si(*p*-C_6_H_4_)_4_ or Si(*p*-C_6_H_4_-C_6_H_4_)_4_ units. This finding indicates that the tetrahedral silicon units are chemically stable and no Si-C bonds have been cleaved during the reaction, in contrary to the possible Si-C cleavage under base conditions (e.g., triethylamine or K_2_CO_3_ as the acid adsorbents) reported in previous literatures [38,42]. These results are in accordance with the ^29^Si NMR results found in silicon-centered POPs [43,44].

### 3.2. Morphology and Thermal Stability

**Figure 4:**
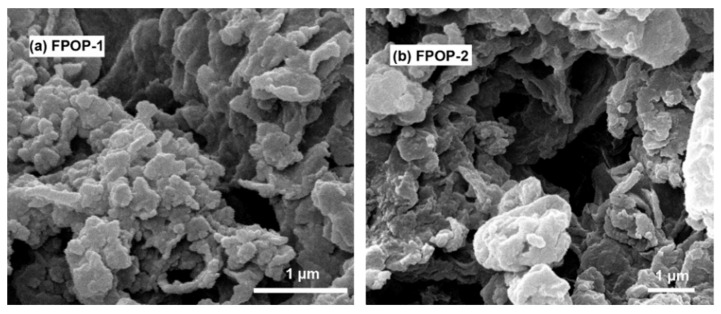
FE-SEM images of FPOP-1 (**a**) and FPOP-2 (**b**).

Figure 4 shows the filed-emission scanning electron microscopy (FE-SEM) images of FPOP-1 and FPOP-2. As expected, these polymers are amorphous and exhibit irregular spherical or bulk particles with the scale at the micrometer sizes.

**Figure 5:**
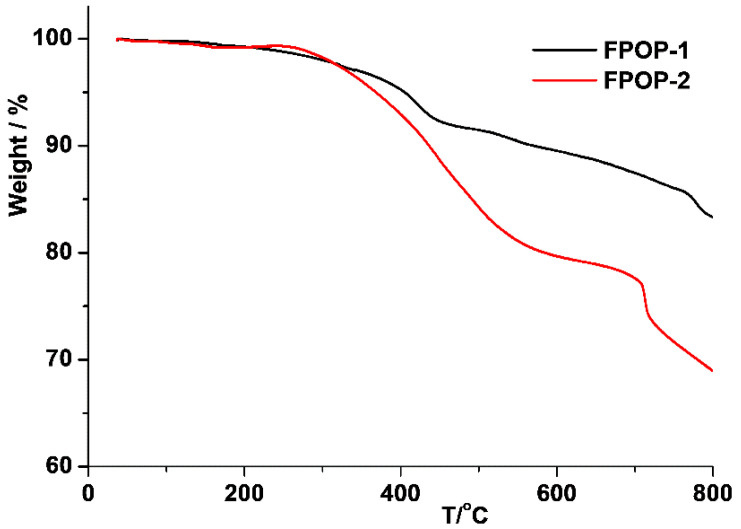
TGA curves of FPOP-1 and FPOP-2 in nitrogen atmosphere.

Figure 5 shows the thermogravimetric analysis (TGA) spectra of these two polymers and the analysis was conducted in an atmosphere of nitrogen at a heat rate of 10 °C min^−1^ ranging from room temperature to 800 °C. Similar to other POP materials [43,44], these polymers also have good thermal stability, while the decomposition temperatures at 5% mass loss (T_d,5%_) were found at 405 °C and 368 °C for FPOP-1 and FPOP-2, respectively. In addition, after the decomposition, the residual ratio of these polymers was higher than 65% at 800 °C. This finding is obviously attributed to their highly cross-linking networks.

### 3.3. Porous Properties

**Figure 6:**
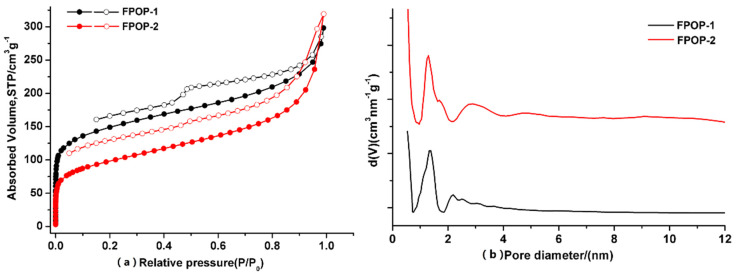
Nitrogen adsorption (close symbols) and desorption (open symbols) isotherms of FPOP-1 and FPOP-2 measured at 77 K (**a**); pore size distribution curves of FPOP-1 and FPOP-2 calculated by NL-DFT (**b**).

The porous properties of these polymers were evaluated by nitrogen adsorption-desorption isotherms at 77 K (Figure 6a) and the data are summarized in Table 1. The isotherms of FPOP-1 and FPOP-2 are similar. Both of them display both type I isotherm and type IV isotherm according to the IUPAC classification [45]. They give rise to a sharp capacity at relatively low pressure and a gradually increasing capacity at relatively higher pressure with hysteresis. This feature indicates that both micropores and mesopores exist within the network. On the basis of the N_2_ isotherm, FPOP-1 has a moderate porosity with the Brunauer–Emmer–Teller (BET) surface area (S_BET_) of 499 m^2^ g^−1^ and the total pore volume (V_total_) of 0.43 m^3^ g^−1^. The micropore surface area (S_micro_) and the micropore volume (V_micro_) were 308 m^2^ g^−1^ and 0.23 cm^3^ g^−1^, respectively, calculated using the *t*-plot method. The V_micro_/V_total_ ratio, which represents the contribution of microporosity in the framework, is 0.53, thereby indicating that mesopores and micropores provide nearly same contribution to the porous network. Compared with FPOP-1, FPOP-2 shows lower porosity with S_BET_ of 354 m^2^ g^−1^ and V_total_ of 0.49 m^3^ g^−1^, and lower microporosity with S_micro_ of 175 m^2^ g^−1^ and V_micro_ of 0.13 m^3^ g^−1^. As well, FPOP-2 possesses a much lower V_micro_/V_total_ ratio of 0.27, thereby indicating that the mesopores mainly contribute to the network. The pore size distributions (PSDs) were estimated by nonlocal density functional theory (NL-DFT). As expected, the PSD results are in accordance with the shape of nitrogen isotherms. FPOP-1 displays two main peaks in the region of micropores centered at ca. 1.4 nm and mesopores centered at ca. 2.1 nm (Figure 6b). The PSD curve of FPOP-2 is similar to that of FPOP-1 except that the mesopore region is broader and centered at 2.8 nm. These results further reveal the co-existence of micropores and mesopores in the porous networks.

Compared to previously reported ferrocene-containing porous polymers, the present polymers exhibit comparable porosity [28,29,46,47]. For example, Yamamoto reactions of 1,1′-dibromoferrocene and 5,10,15,20-tetrakis(4-bromophenyl)porphyrin or tetra(*p*-bromophenyl)methane resulted in two ferrocene-based conjugated microporous polymers (CMPs) with the S_BET_ of 638 m^2^ g^−1^ and 422 m^2^ g^−1^ [31]. We prepared a ferrocene-based POP with the S_BET_ of 542 m^2^ g^−1^ through the Sonogashira reaction of 1,1′-dibromoferrocene and tetrakis(4-ethynylphenyl)silane [48]. In addition, FPOP-1 exhibits higher porosity than FPOP-2. This finding may be due to the different structure length of these two silicon-centered monomers. It was known that longer monomer structure length could result in lower surface areas due to greater interpenetration and more efficient space filling in the network, which was induced by increased degree of conformational freedom [49]. In the present study, tetrakis(4′-bromo-[1,1′-biphenyl]-4-yl)silane has a longer structure length than tetrakis(4-bromophenyl)silane, thereby resulting in lower porosity.

### 3.4. Carbon Dioxide Sorption

**Figure 7:**
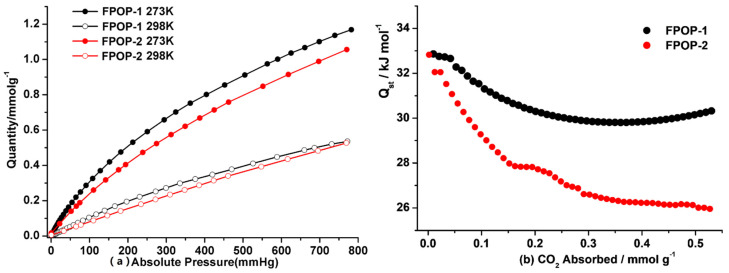
CO_2_ adsorption isotherms of FPOP-1 and FPOP-2 at 273 and 298K (**a**); isosteric heat of CO_2_ adsorption of FPOP-1 and FPOP-2 (**b**).

One important application of the porous materials is gas sorption. Herein, carbon dioxide (CO_2_) was selected as a representative gas because CO_2_ emission has played an important role in global warming and become a major concern for environment [1,50]. Moreover, the capture of CO_2_ is important and highly beneficial to reach net-zero emissions in the second half of the century [51]. Various porous materials, including zeolites, porous carbon, and metal-organic frameworks, have been applied as adsorbents for the capture or storage of CO_2_ in addition to other efficient techniques, such as membrane separation, chemical adsorption, and gas hydrates [52,53,54]. Among various porous materials, POPs are particularly promising thanks to their high porosity and facile tunability of their pore surface functionality (e.g., ferrocene units in the networks), which can play an important role in improving the interactions with CO_2_. Therefore, the CO_2_ adsorption ability of FPOP-1 and FPOP-2 was evaluated. The isotherms are shown in Figure 7 and the data are summarized in Table 1.

FPOP-1 possesses moderate CO_2_ capacities of 1.16 mmol g^−1^ (5.1 wt%) at 273 K and 1.0 bar, and 0.54 mmol g^−1^ (2.38 wt%) at 298 K and 1.0 bar. Compared to FPOP-1, FPOP-2 with a lower surface area expectedly displays lower CO_2_ capacity, which was found to be 1.06 mmol g^−1^ (4.66 wt%) at 273 K and 1.0 bar, and 0.52 mmol g^−1^ (2.29 wt%) at 298 K and 1.0 bar (Figure 7a). Although the values are not very high, they are comparable to some polymers having similar or much larger specific surface areas, such as triazine-containing CMP (TCMP-5, S_BET_: 494 m^2^ g^−1^, 1.22 mmol g^−1^ at 273 K/1 bar) [55], CMP-5 (S_BET_: 494 m^2^ g^−1^, 1.22 mmol g^−1^ at 273 K/1 bar) [55], CMP-1-(NH_2_) (S_BET_: 710 m^2^ g^−1^, 1.56 mmol g^−1^ at 273 K/1 bar), and COF-102 (S_BET_: 3620 m^2^ g^−1^, 1.56 mmol g^−1^ at 273 K/1 bar) [56]. The interactions between the polymer framework and CO_2_ molecules were further evaluated by calculating the isosteric heats (Q_st_) while employing the Clausius–Clapeyron equation from CO_2_ isotherms at 273 K and 298 K. The Q_st_ values of FPOP-1 and FPOP-2 are 32.9 kJ mol^−1^ and 32.5 kJ mol^−1^ at low CO_2_ coverage, and gradually decrease to ca. 30 kJ mol^−1^ and 25 kJ mol^−1^ with the adsorption increasing at higher coverage, respectively (Figure 7b). These findings reveal that the polymer frameworks possess strong binding ability with CO_2_ because of the presence of the ferrocene unit as a withdrawing electron group, which can strongly interact with electron-rich CO_2_ molecules [24]. In addition, the values of Q_st_ are comparable or higher than previous ferrocene-based POPs [44,47,48]. These results reveal that these polymers can be applied as efficient adsorbents for CO_2_ capture and storage.

## 4. Conclusions

Two ferrocene-containing porous organic polymers (FPOPs) were synthesized based on 1,1′-divinylferrocene and tetrahedral silicon-based monomers, i.e., tetrakis(4-bromophenyl)silane and tetrakis(4′-bromo-[1,1′-biphenyl]-4-yl)silane via the Heck reaction. The resulting materials exhibit high thermal stability and comparable porosity with the highest BET surface area of 499 m^2^ g^−1^ and total pore volume of 0.49 cm^3^ g^−1^. The porosity is comparable to previous ferrocene-based POPs. For applications, these polymers display CO_2_ uptakes of 1.16 mmol g^−1^ (5.10 wt%) at 273 K and 1.0 bar, and 0.54 mmol g^−1^ (2.38 wt%) at 298 K and 1.0 bar (FPOP-1), which are comparable to many POP materials with similar or higher surface areas. Moreover, they both possess high isosteric heats of up to 32.9 kJ mol^−1^, indicating their high affinity towards CO_2_ due to the presence of ferrocene units in the porous networks. These results demonstrate that these materials can be promisingly used as adsorbents for the storage and capture of CO_2_. Further investigation will focus on the construction of ferrocene-based POPs with high surface areas by altering different aromatic monomers to react with 1,1′-divinylferrocene.

## Figures and Tables

**Table 1:** Porosity data of FPOP-1 and FPOP-2.

	S_BET_^(a)^/m^2^ g^–1^	S_micro_^(b)^/m^2^ g^–1^	V_total_^(c)^/cm^3^ g^–1^	V_micro_^(d)^/cm^3^ g^–1^	V_micro_/V_total_	CO_2_ Uptake (wt%) ^(e)^
FPOP-1	499	308	0.43	0.23	0.53	5.10
FPOP-2	354	175	0.49	0.13	0.27	4.66

^(a)^ Surface areas calculated from N_2_ adsorption isotherm using the BET method; ^(b)^ microporous surface areas calculated from N_2_ adsorption isotherm using the *t*-plot method; ^(c)^ total pore volumes calculated at P/P_0_ = 0.90; ^(d)^ microspore volumes derived using the *t*-plot method based on the Halsey thickness equation; ^(e)^ carbon dioxide uptakes at 1.0 bar and 273 K.

## Data Availability

The data presented in this study are available upon request from the corresponding author.
